# Genome Sequencing and Assembly by Long Reads in Plants

**DOI:** 10.3390/genes9010006

**Published:** 2017-12-28

**Authors:** Changsheng Li, Feng Lin, Dong An, Wenqin Wang, Ruidong Huang

**Affiliations:** 1 College of Agronomy, Shenyang Agricultural University, 120 Dongling Road, Shenyang 110866, China; lcslyh@yahoo.com; 2 College of Bioscience and Biotechnology, Shenyang Agricultural University, 120 Dongling Road, Shenyang 110866, China; fenglinsn@126.com; 3 School of Agriculture and Biology, Shanghai Jiao Tong University, 800 Dong Chuan Road, Shanghai 200240, China; dongan1983@sjtu.edu.cn

**Keywords:** genome assembly, long reads, Sanger sequencing, Next Generation Sequencing, Third Generation Sequencing

## Abstract

Plant genomes generated by Sanger and Next Generation Sequencing (NGS) have provided insight into species diversity and evolution. However, Sanger sequencing is limited in its applications due to high cost, labor intensity, and low throughput, while NGS reads are too short to resolve abundant repeats and polyploidy, leading to incomplete or ambiguous assemblies. The advent and improvement of long-read sequencing by Third Generation Sequencing (TGS) methods such as PacBio and Nanopore have shown promise in producing high-quality assemblies for complex genomes. Here, we review the development of sequencing, introducing the application as well as considerations of experimental design in TGS of plant genomes. We also introduce recent revolutionary scaffolding technologies including BioNano, Hi-C, and 10× Genomics. We expect that the informative guidance for genome sequencing and assembly by long reads will benefit the initiation of scientists’ projects.

## 1. Introduction

Plant genomes contain important information for understanding their architecture. The genome sequences facilitate the study of plant comparative genomics and also serve as a valuable resource for the research of plant flowering evolution. High quality reference genome assemblies are critical in accelerating plant breeding by selecting desirable genes with improved agronomic traits, including high yield, tolerance to various environmental stresses, and resistance to pathogens. During plant evolution, the genome is reshaped by active transposon amplification [1]. Long terminal repeat (LTR) retrotransposons are the most prevalent elements in plant genomes that amplify by using a “copy-and-paste” mechanism. Their length usually exceeds 10 Kb leading to incomplete and fragmented assembly. To increase crop yield and nutrition in the process of domestication, the gene content may have been enlarged by gene family expansion, whole genome duplication, and polyploidy. For example, the gene families involving flower and fruit development in domesticated apples have been expanded [2]. The specific flavonoid-related gene family is found to have expanded in cacao [3]. Alpha zein protein comprises the majority of seed storage proteins in maize endosperm, which have more than 40 copies spreading over six loci with a size of 30–500 Kb in inbred maize [4]. The high similarity of paralogs from gene families creates ambiguities during genome assembly where two or more homologous regions assemble. Whole genome duplication (WGD) happens in most plant genomes, including sorghum, rice, and cucumber etc. A relatively recent WGD in apples happened 50 million years ago [2]. A duplicated chromosomal segment in *Sorghum* appears to be only a few million years old [5], indicated by the high similarity of the sequences. Comparative studies show that repetitive sequences, transposable elements, and gene duplication have significantly proliferated in gymnosperms [6]. The largest genome, that of the 22-Gb loblolly pine, has presented formidable technical challenges for whole-genome shotgun sequencing and assembly [7].

With the advent of Next Generation Sequencing (NGS), we are facing an explosion of released genomes, such as loblolly pine, cotton, pear, and pepper [8]. However, draft genomes are suffering from unknown sequences and ambiguous assembly due to homologous sequences, whereas high-quality genomes are demanded for comparative genomics and functional annotation to improve agronomic traits in plant breeding. Third Generation Sequencing (TGS) produces reads with a mean length of 20 Kb and a maximum length of 60~200 Kb [9,10,11]. Long-read sequencing is a great tool to overcome the low resolution of reconstructing repetitive regions and polyploidy. In this review, we focus primarily on the advancement and limitations of TGS in plant genome assembly and annotation. We aim to give readers guidance and considerations before their project initiation. 

## 2. Sanger Sequencing: A Milestone in Plant Genomics

Sanger sequencing is a method of DNA sequencing based on the selective incorporation of chain-terminating dideoxynucleotides in the process of DNA amplification. It is accurate but low throughput with a sequencing length of 800 bp (Table 1). A set of ‘gold standard’ reference genomes for *Arabidopsis* [12], rice [13], *Sorghum* [5], and maize [14] were first sequenced using reiterative Sanger-based approaches along the minimal tiling path of bacterial artificial chromosomes (BACs). Chromosome 4 of the rice genome (*Oryza sativa*) was constructed with 287 BACs and two phage (P1)-derived artificial chromosomes. Each clone was sequenced by a random shotgun approach with tenfold coverage [15]. The first version of the maize genome was shotgun-sequenced with 4- to 6-fold coverage with BAC by BAC (*n* = 16,848) by using a minimum tiling path derived from an integrated physical and genetic map [14]. Still, genome sequencing from BAC clones with large inserted DNA fragments is non-trivial work in terms of time and expense, which includes constructing more than ten times the coverage of the library, developing physical maps from the pattern of shared restriction fragments, and selecting a minimal tilling path.

## 3. Next Generation Sequencing Enables Unprecedented Development in Plant Genomics

Next Generation Sequencing, also known as high throughput sequencing, can produce unprecedented data, enabling researchers to study both genomics and transcriptomics with read lengths of up to 300 bp (Table 1). The massive increases in data volume and greatly improved accuracy make NGS economical in sequencing most plant genomes. The number of sequenced plant genomes has exploded to ~200 species, from model to non-model species [8,9]. The statistics of published plant genomes and how they are sequenced (Sanger only, or Illumina only, or the hybrid) are extensively reviewed in [8]. The high throughput and low cost of NGS technologies facilitates the sequencing of several small crop genomes less than 500 Mb, including cucumber [16], apple [2], wild strawberry (*Fragaria vesca*, diploid) [17], cacao [3], and date palm [18], providing invaluable genomic resources for vegetable and fruit breeding.

NGS also promotes sequencing multiple cultivars of the same species. A sequencing project of 360 wild and cultivated tomato accessions discovered that two potential quantitative trait loci (QTLs) increased fruit size 100-fold during tomato domestication [19]. Resequencing of 20 watermelon accessions constructed the genetic diversity and population structure of watermelon germplasms, and also identified the genes responsible for fruit quality traits [20]. To discover allelic variants and improve rice production, a more ambitious project of resequencing 3000 rice accessions from 89 countries has been conducted [21]. They found large-scale genomic diversity, serving as a great foundation for the discovery of novel alleles for important rice phenotypes. With the release of the sequencing data, the global rice community can take advantage of this data to accelerate rice breeding and improvement [21].

Different from mammalian genomes, plants have high repeat content and variable genome sizes. *Genlisea tuberosa* bears the smallest genome of 61 Mb, while the wheat genome is 17 Gb with 90% repetitive sequences [8]. The loblolly pine genome (22 Gb) is the largest published genome to date [7]. However, the short reads cannot fully span over the repetitive regions and resolve polyploidy, resulting in tens of thousands of fragmented assemblies and collapsed contigs. The short-read lengths of NGS with inherent biases lead to incomplete genomes, where the missing sequences could be biologically informative including entire genes, regulatory elements and repeat elements of transposable elements (TEs), centromeres, and telomeres.

Long terminal repeats are identical sequences of DNA with hundreds or thousands of copies in plant genomes that are composed of two groups: Copia and Gypsy. Copia elements have a length of 4–8 Kb and Gypsy elements are 8–16 Kb [22,23]. In comparison to retrotransposons, there are numerous copies of CACTA elements in cereal genomes that can have more than 3000 copies with a length of more than 10 Kb [24]. For example, TEs represented 84.4% of the genome sequence of the progenitor of the wheat D genome *Aegilops tauschii*, while long terminal repeat retrotransposons, such as Gypsy and CACTA super-families, were dominant in the genome with the ratio of 65.9% [25]. Short-read sequencing could not effectively resolve the repeat regions, leaving the missing sequences, called gaps or unordered short contigs in the assemblies. Only the long reads can overcome the obstacles because they can jump over the gaps by connecting two contigs into a scaffold with a length of 10–20 Kb. Thanks to TGS, it offers the opportunity to crack complex plant genomes.

## 4. Long-Read Sequencing Opens a New Era of Solving Complex Plant Genomes

### 4.1. TruSeq Synthetic Long-Read

A superior way to resolve transposon repeats is to generate long reads that exceed transposon regions, providing unique flanking sequences to span over the ambiguous locations. A novel technology introduced by Illumina is the TruSeq Synthetic Long-Read (SLR) using highly-parallel library preparation and DNA barcode kits, which allows for the construction of synthetic long reads from the short sequencing reads generated with its existing HiSeq platform. The reported length can be 1.5–18.5 Kb with an accuracy of 99.9% [26]. SLR offers a powerful method for complex and repeat-rich genomes compared with Illumina short reads. The assembled genome with synthetic long reads for the model organism *Drosophila melanogaster* obtains a contig N50 of 69.7 Kb and covers 96.9% of the current reference genome. At least 40 Kb of missing genomic sequences was found in the *Caenorhabditis elegans* genome using long reads [27]. The length of the assembled DNA molecule was also increased for the large 725 Mb genome of *Botryllus schlosseri* using the same approach [28]. Still, the assemblies hardly reach a contig N50 more than 100 Kb despite the high quality of SLR.

### 4.2. Nanopore Sequencing

Oxford Nanopore sequencing and PacBio single-molecule real-time (SMRT) sequencing are the most popular platforms in the market that produce long reads. Both platforms generate sequence reads of up to tens of thousands of bases without artificial amplification, but has a relatively high error rate when compared to Illumina. The DNA sequence in Oxford Nanopore sequencing is determined by the variation from differently labelled nucleotides when the DNA string goes through a tiny nanopore in a flowcell. Thus, the theoretical read length is only limited by the length of the DNA molecule. A complete bacterial genome was assembled de novo in a single 4.6 Mb contig using only Nanopore sequencing data [29]. The hybrid of Nanopore reads and MiSeq data produced a highly contiguous and accurate fungal genome for *Saccharomyces cerevisiae*. The contig N50 length is ten times longer than an Illumina-only assembly (678 Kb versus 59.9 Kb), presenting a much more complete representation of the features of repeats and elements that are absent in Illumina-only assemblies [30]. Recently, Nanopore sequencing began to sequence complex genomes of animals and plants. The 860 Mb genome of the endangered European eel was sequenced using Oxford Nanopore sequencing. The resulting genome assembly gained an N50 contig of 1.2 Mb, significantly improved from the previous version from short reads only [31]. 91.2 Gb of sequence data (~30× theoretical coverage) from 39 flowcells was generated for the human genome, leading to a highly complete and contiguous assembly with a N50 contig of ~3 Mb. The data permits sensitive detection of both large structural variants and epigenetic modifications in humans [32]. A new *Solanum pennellii* accession was de novo assembled by using Nanopore sequencing with a median read length of 11,979 bp. A contig N50 of 2.5 Mb was achieved for the 1.2 Gb genomes structurally similar to that of the reference tomato [33]. The genome of *A. thaliana* sequenced by an Oxford Nanopore MinION sequencer was assembled into 62 contigs with an N50 length of 12.3 Mb, covering 100% (119 Mb) of the non-repetitive genome. Noticeably, Nanopore sequencing was able to assemble the ribosomal DNA (rDNA) and centromeres in *Arabidopsis* because it can produce reads of up to 200 Kb [34]. It is promising that Nanopore sequencing has the potential to make high-quality genomic resources. However, so far there are few reports on plant genome assemblies and it is still at an incipient stage [9].

### 4.3. PacBio Sequencing

Another long-read technology is SMRT sequencing, which takes advantage of the natural process of DNA replication and enables real-time observation of DNA synthesis by incorporation of zero-mode waveguides (ZMWs) and phospholinked nucleotides. Each step of template extension generates a light pulse that can be recognized as a differently labelled nucleotide. Currently, the average read length could be over 10 Kb by using P6C4 chemistry for PacBio (Table 1). The newly released Sequel System claims to target longer reads and higher throughput with cheaper sequencing costs. The long reads allow for spanning of repeat regions, resolving many repetitive sequences and assisting genome reconstruction. With the increased read length and decreased errors, long-read sequencing has greater applications in plant science, even for large and complex genomes. PacBio sequencing generated 424 contiguous and non-chimeric wheat storage protein transcripts, opening a way for studying gene amplification and copy number variation among species and cultivars [35]. The six loci containing tandem zein gene copies were reconstructed in W22 inbred maize by using single-molecular real-time sequencing, skipping the process of sequencing the overlapping BAC clone, which is expensive and labor-intensive [4]. Several high-quality genomes have been published with improved continuity and accuracy, including *Utricularia gibba* (82 Mb), *Oropetium thomaeum* (245 Mb), *Chenopodium quinoa* (1500 Mb), *Zea mays* (2300 Mb), and *Helianthus annuus* (sunflower, 3000 Mb) (Table 2).

## 5. Application and Consideration of PacBio Sequencing

Genome sequencing by long reads has exhibited promising applications in three fields: de novo assembly, scaffolding, and gap-filling. Considering SMRT sequencing from PacBio is the most-widely used platform, we will focus specifically on PacBio sequencing here for long-read genome assembly.

### 5.1. Application of PacBio Sequencing

#### 5.1.1. De Novo Assembly

Long-read sequencing is useful to de novo assemble genomes. A de novo assembly of *O. thomaeum* generated a contig N50 of 2.4 Mb with a genome coverage of 99% (244 Mb). The completeness of the *Oropetium* genome captures all 18 telomeric arrays and nine centromeric satellites that are often unassembled in most plant genomes [36]. A quinoa genome assembly contained 3486 scaffolds, with a scaffold N50 of 3.84 Mb, while 439 scaffolds covered 90% of the assembled genome [37]. *H. annuus* genome sequencing from long reads generated a genome assembly that captured 3 Gb being 80% of the estimated genome size of 3.6 Gb. We can see that such contiguity from the assembly of long reads is never reached by short reads. In addition, the contigs contain fewer gaps, featuring better completeness of genome coverage compared to short-read assemblies. For example, the genome assembly of *Arabis alpine* from PacBio reads generated 30 Mb more sequences than the one from short reads, but the gap ratio of Ns (unknown sequences) was reduced from 9.2% to 3.3% [38].

#### 5.1.2. Scaffolding

The initial genome assemblies based on Sanger sequencing or NGS are composed of many small contigs, some of which are arbitrarily ordered and oriented, markedly complicating and impeding investigation of causative loci and phenotypic traits. The long reads from PacBio provide frameworks to scaffold genomes, resolve ambiguities, and reorder gene orientation. The improved version of the maize genome assembly is made up of 625 scaffolds in Version 4 rather than 61,161 ones in Version 3. The contig N50 is more continuous (1180 Kb) in Version 4 than Version 3 (19 Kb) (Table 3). Several previously identified megabase­sized mis­oriented pericentromeric regions were corrected in the genome of Version 4 [10,14]. The long-read assembly is of 122-fold higher contiguity than the recently published short-read genome assembly of *U. gibba* (contig number: 581 versus 3843; contig N50: 3.4 Mb versus 28 Kb) [39,40]. Still, the costs of PacBio sequencing are quite substantial compared to NGS. To compromise, a couple of genomes were assembled from short reads only, then lower amounts of long reads were used to improve genome assembly by gap closure or scaffolding. The Petunia genome and the parents were assembled by Illumina reads, re-scaffolded, and gap-filled using PacBio reads [41]. The integration of PacBio long reads with Illumina paired-end short reads obtained a high-contiguity genome assembly of the complicated allopolyploid genome of *Brassica juncea* [42].

#### 5.1.3. Filling the Gaps

The assembly could make substantial improvements for gaps in plant genomes, even in repeat-rich regions, centromeres, and telomeres. The percentage of missing sequences is greatly reduced from 10% to 3% of the maize reference genome, although there are still 2522 gaps. Most centromeres and telomeric repeats are accurately placed and largely intact. In addition, the improved assembly also increases the coverage of regulatory sequences, enhances the annotation, and raises our ability to identify functional genetic variation [10]. The highly contiguous genome assembly allows for 7.7% more annotated protein-coding gene than reported from short-read assembly. The alignment of the short-read assembly against the long-read assembly for *U. gibba* demonstrates that most of the DNA gained by PacBio sequencing contains repeated elements, particularly surrounding putative centromeres [40].

### 5.2. Consideration of PacBio Sequencing

#### 5.2.1. Sample Preparation

**Figure 1:**
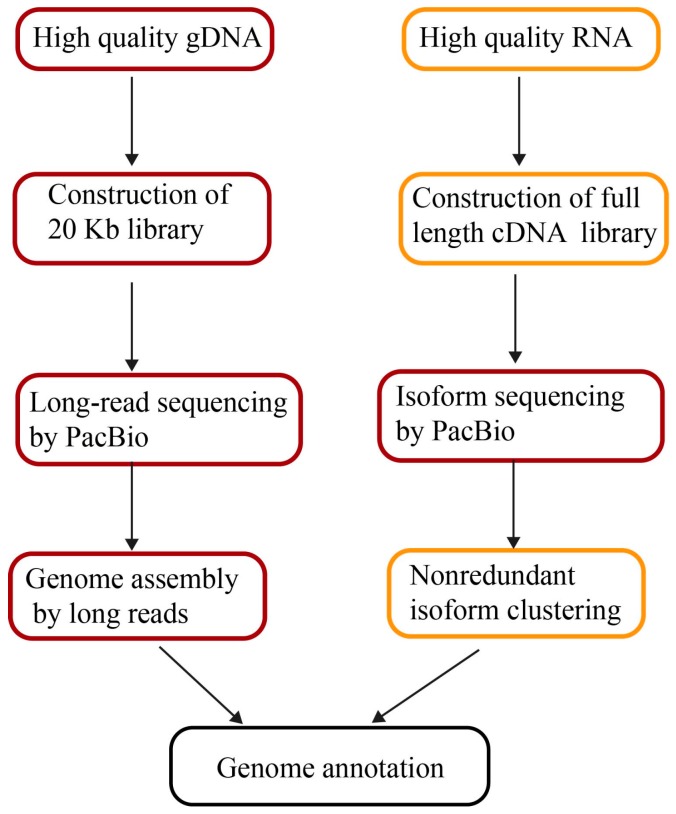
The pipeline of genome assembly and annotation by long reads. gDNA: genomic DNA; cDNA: complementary DNA.

Structural heterozygosity in genomes results in separate contigs. The DNA samples from inbred lines or double haploid strains are always the priority to minimize heterozygosity. Long-read sequencing by PacBio does not require temperate amplification. The extracted DNA is directly used as templates in library preparation. Any irreversible DNA damage present in the input material leads to poor quality DNA sequencing. Thus, the integrity, purity, and concentration of genomic DNA is imperative for obtaining long-read lengths and assembling quality genomes afterwards (Figure 1). There are two methods to extract high-quality DNA in plants. One is to prepare a ~20 Kb SMRTbell library following the PacBio protocol [43], for instances of maize [10] and quinoa DNA preparation [37]. Another is to precipitate DNA from intact nuclei [44]. Isolated nuclei were collected from a density gradient, after which high-molecular-weight DNA was isolated from the nuclei of *U. gibba* plants [40]. Fifty micrograms of high-molecular-weight *Oropetium* genomic DNA (gDNA) was prepared by using a modified nuclei preparation method followed by additional high-salt phenol-chloroform purification to minimize contamination [36].

#### 5.2.2. Sequencing Strategy

The sequencing strategy depends on project objectives, genome size, and complexity, as well as the quality and type of data available. In general, for de novo assembly, more than 50 times coverage is necessary. The published genomes from long-read assemblies all contain deep coverage, for instance, 65× of *Z. mays* and 102× *H. annuus*. To improve existing assemblies, more than 10 times coverage of long-read sequencing is required for scaffolding and gap filling. The read length is critical for successful assembly and to maximize potential performance. The 20 Kb insert libraries combined with two runs of size selection are also beneficial for increasing read lengths (Figure 1). The mean lengths of long reads in the high-quality genomes of *Oropetium*, quinoa, maize, and *H. annuus* are all more than 10 Kb (Table 2).

#### 5.2.3. Bioinformatics Analysis

A couple of algorithms have been developed to improve genome assembly. The hybrid solution combining long reads with short reads is efficient for de novo assembly as the error-prone base calling of PacBio is corrected by Illumina reads, whereas at least two libraries are required for preparation. The corrected long reads achieve >99.9% accuracy, leading to substantial assembly improvement [45,46]. When the sequence coverage is high enough, the long reads can correct themselves without short-read correction anymore. The method, called Hierarchical Genome Assembly Process (HGAP), firstly uses the longest reads as seeds to recruit all other reads for the construction of highly accurate preassembled reads, secondly assembles the preassembled reads using a Celera Assembler, then polishes using Quiver. HGAP is adapted to assemble hundreds of Mb-sized genomes by only using PacBio long reads, such as in bacteria [47] and small genome sized plants [36,40]. For instance, it was applied to assemble the 82 Mb carnivorous plant genome of *U. gibba* with a retrieved contig N50 of 3.4 Mb [40]. It was also used in the assembly of desiccation-tolerant grass *O. thomaeum*, producing a contig N50 of 2.4 Mb [36]. Falcon is a de novo genome assembler. The long-read sequence assembly of the gorilla genome using Falcon generates a contig N50 of 9.6 Mb [48]. Falcon-Unzip is also a diploid-aware assembler. It takes the contigs from assembly and phases the reads according to heterozygous single nucleotide polymorphisms (SNPs) into a haplotype-resolved assembly. The heterozygous genomes of the F1 hybrid of *A. thaliana*, the highly heterozygous outcrossed grape cultivar of *Vitis vinifera* and the fungus of *Clavicorona pyxidata*, resist assembly by short reads. They are sequenced by PacBio long reads and assembled by Falcon-Unzip algorithms, which are more contiguous and complete than other approaches. The phased diploid assembly enables the identification and study of heterozygous structural variations in homologous regions [49].

However, one tool cannot fit all genomes. The Falcon assembler was tested to assemble 250 Mb *O. thomaeum*. It was found that the Falcon assembler has lower contiguity than HGAP assembly and has fewer assembled centromeres and telomeres [36]. The maize genome containing 2300 Mb was assembled with Falcon and PacBio Corrected Reads Hierarchical Assembly Pipeline (PBcR)). Given the fewest conflicts with PBcR, the assembly was adopted as the new B73 genome reference with a contig N50 of 1.1 Mb [10]. More customized algorithms and pipelines are created. A three Gb *H. annuus* genome was assembled by a pipeline of PBcR to error correct reads, WGS to assemble, and Quiver to polish the consensus sequence [11]. The 1500 Mb quinoa genome was conducted by using the SMRT-make assembly pipeline [37]. The Masurca assembler integrates the benefits of the *deBruijn* graph and Overlap-Layout-Consensus assembly approaches, which supports hybrid assembly with short Illumina reads and long but error-prone PacBio/Nanopore sequences [50]. The large and extremely repetitive plant genome of *A. tauschii* has impeded assembly attempts only by short reads. The technique of long-read sequencing by PacBio corrected by accurate short Illumina reads was performed to produce mega-reads, which were assembled into contiguous contigs with an N50 contig size of 487 Kb [25,51]. Canu, a successor of the Celera Assembler [52], is specialized in assembling the high error rates of PacBio or Nanopore sequencing [53]. Canu can auto-detect computational resources and scale itself, greatly improving the efficiency of big genome assembly. It is reported that Canu can reliably assemble complete microbial genomes and near-complete eukaryotic chromosomes, achieving a contig NG50 of greater than 21 Mb in both humans and *D. melanogaster* [53].

## 6. Long-Range Scaffolding Technologies Improve Assembly

It is almost impossible to assemble a complete genome from sequence reads alone due to redundant repeats and the complexity of plant genomes. After we get contigs from sequence assembly, we typically order contigs into scaffolds using alignments of paired reads from BAC or fosmid ends. Furthermore, to reach chromosome-level contiguity, additional genetic or physical/optical maps are still required. It is worth mentioning that the high-throughput technologies of physical mapping, such as BioNano, Chromosome Conformation Capture (Hi-C), and 10× Genomics have emerged, whereas traditional genetic mapping or physical mapping is labor intensive and time-consuming. 

Complementary to DNA sequencing technologies, BioNano mapping can help scaffold genome assemblies by using linkage information from the physical location of restriction enzyme digestion. BioNano mapping can identify assembly errors, anchor scaffolds, and improve the contiguity of draft genome assemblies. For example, chromosome-scale assembly was generated with a scaffold N50 of 3.84 Mb in quinoa and of 7.8 Mb in *O. thomaeum* when combined with BioNano mapping [36,37]. The assembly of 2958 contigs from long reads in the maize reference genome is reduced to 625 scaffolds with the integration of an optical map by BioNano mapping [10]. Optical mapping can even identify structural variations. The comparative BioNano optical mapping of two inbred maize lines W22 and Kill revealed a prevalence of deletions in regions of low gene density and maize lineage-specific genes [10].

Another technique to develop chromosome-scale assembly involves capturing the conformation of genomes. This method is based on Hi-C, in which chromatin is crosslinked, digested, and re-ligated in such a way that only the DNA fragments that are covalently linked together form ligation products. The ligation products are subject to deep sequencing, giving their physical information in the genomic sequence [54]. The combination of sequence assembly and Hi-C data greatly improves the contiguity of genomes. The barley genome is characterized by abundant repetitive sequences, leading to limitations in the contiguity of whole-genome assembly. The technique of Hi-C was applied to order the reference sequence of the barley genome, especially across the pericentromeric region at megabase resolution [55]. Three relatives of the model plant *A. thaliana* were sequenced with PacBio long-read data and assembled into a few hundred contigs. After using optical mapping and chromosome conformation capture data, the lengths of scaffolds doubled those of contigs and the misassembled contigs were corrected [56]. It is obvious that Hi-C is targeted to link DNA fragments within a distance of more than hundreds of Kb, whereas the proximity is limited. Dovetail Genomics [57], another long-range scaffolding technology dedicated to simplifying genome assembly, can link DNA pieces that are 10~100 Kb away from each other with increased resolution. The technology integrates high-quality long-range genomic information with NGS sequencing. The simple approach helped scaffold a human genome with a scaffold N50 of 20 Mb and an American alligator genome to 10 Mb [58].

The last long-range scaffolding technology highlighted here is 10× Genomics. It is an assembly solution using a barcode group of linked short reads that originate from the same individual DNA molecule [23]. Seven human genomes were de novo assembled with low-cost HiSeq× data and the 10× Genomics approach, yielding a contig N50 of more than 100 Kb and a scaffold N50 of 20 Mb [59]. High-throughput linked-read sequencing reconstructs haplotypes of human chromosomes and detects genetic variation [60]. 10× Genomics greatly reduces sequence costs and improves assembly contiguity simultaneously by using short-read library preparation methods to extend scaffolds. Still, it lacks the fine resolution to improve contig lengths directly in contrast to PacBio or Nanopore sequencing. So far, the technology of 10× Genomics has not been used in any published plant genome [60].

The quality of the completeness and contiguity of a genome can be evaluated by the parameters of contig N50, scaffold N50, and genome coverage. Contig N50 is the shortest contig length in the list of half of the total length of all assembled contigs. With other information from physical and genetic maps, contigs can be further merged into scaffolds. It is well accepted that the better assembly is that with the longer contig and scaffold N50. Genome coverage refers to the percentage of the genome that is included in the assembly. Genome coverages of 90–95% are generally considered to be good due to the repeats that are difficult to sequence and order. A new set of standards has been defined for categories of genome assemblies, including standard draft, high-quality draft, noncontiguous finished, and finished. Finished quality standards, commonly known as the Bermuda standards, define a finished sequence as a contiguous sequence with less than one error per 10,000 bases [61]. Overall a high-quality draft is required to be a better target for annotation [62]. It is almost impossible to get full genomes due to the abundant repetitive regions that are difficult to assemble in plants [62]. Compared with Sanger sequencing and NGS, genome assemblies from long reads have been greatly improved. The contig N50 in the latest version of the maize genome is 1180 Kb compared with the last version of 19 Kb, equal to 62-times longer (Table 3). The scaffold N50 is significantly increased from 76 Kb to 9.5 Mb. The missing regions are reduced to 3% from 10% by gap-filling of repeats, telomeres, and centromeres (Table 3) [10].

## 7. Genome Annotations by Long Reads

Following genome assembly, the determination of gene structure and assignment of gene function are still required. Genomes can be annotated by expressed sequence tags (EST), protein sequences from related species, and an ab initio approach involving computational training and gene finding. RNA sequencing (RNA-seq) data have the greatest potential to improve the accuracy of gene annotations, as these data provide copious evidence for better delimitation of exons, splice sites, and alternatively spliced exons. RNA-seq reads can be de novo assembled and then realigned to the genome in the same way as ESTs. Alternatively, RNA-seq data can be directly aligned to a genome, followed by the assembly of alignments (rather than reads) into transcripts that need a reliable reference genome. 

However, EST and RNA-seq do not provide full-length transcript sequences, confounding their application for defining alternative splicing. Furthermore, it is challenging to identify nearly identical gene family members with misassembled transcripts, leading to incorrect annotations [63]. Full-length transcripts can readily facilitate the accuracy of genome annotation because full-length transcripts permit efficient dissection of the structure of exons and introns, as well as alternative splicing. The third-generation sequencing technology of PacBio offers a unique opportunity for constructing full-length transcripts directly with long reads of up to 20 Kb, called isoform sequencing (Iso-Seq). Full isoform sequencing facilitates unveiling the complexity of transcriptomes via defining novel genes and splicing in plants (Figure 1). In wheat, a total of 91,881 full-length transcripts were identified as 13,162 known genes and 3026 new genes that had not been previously annotated. The elucidation of 72 transcribed members with full-length transcripts from the gluten gene family is important for common wheat breeding [64]. In maize, 111,151 transcripts produced by Iso-Seq from six tissues retrieved ~70% of the annotated genes in the maize genome. 57% of the transcripts represent novel or tissue-specific isoforms of known genes and 3% correspond to novel gene loci [63]. The valuable full-length transcripts not only improve maize genome annotation but also enhance our understanding of the complex transcriptome network.

## 8. Future Perspectives

Long-read sequencing provides unprecedented opportunities for plant genomics both in assembly and annotation. The resolution of repetitive regions is highly improved due to the spanning of long reads over the repeats. With the complementation of optical maps, assembled genomes are validated and further scaffolded. Long reads of isoform sequencing offers new solutions for finding new genes and variable splicing. We will gain deeper understanding of genomic diversity, evolution, and gene function by long-read sequencing, thus in turn accelerating the process of plant breeding and speeding up the production of improved varieties. 

What happens when a sequencing read has no length limit? What if we can sequence one chromosome in just one read? We are still waiting for the time point where the sequence reads can be long enough to be uniquely distinguished, and when a complete plant genome is trivial to obtain at low cost. At that time, this will enable powerful functional studies and exploration of comparative evolution, which will in turn speed up plant research. We believe that ongoing technology advances will override length-limit obstacles. In very recent times, Nanopore sequencing can sequence up to one Mb which is not limited by technology but by library preparation [33].

## Figures and Tables

**Table 1:** Comparisons and summary of sequencing technologies.

Categories	1st Generation	2nd Generation					3rd Generation	
Platform	Sanger	Illumina					PacBio	Nanopore
		HiSeq2500–High output	HiSeq2500–Rapid mode	MiSeq	Synthetic Long reads	10× Genomics		
Read length	800 bp	2 × 125 bp	2 × 250 bp	2 × 300 bp	~100 Kb	up to 100 Kb	10−15 Kb	up to 200 Kb
Yield/Cell	80 Kb	450−500 Gb	125–150 Gb	13–15 Gb	See HiSeq2500	See HiSeq2500	5–10 Gb	up to 1.5 Gb
Instrument Time	3 h	6 days	60 h	21–56 h	See HiSeq2500	See HiSeq2500	4 h	2 days
Price/Gb	$1,000,000	$30	$40	$110	$1000	See HiSeq2500 + $500/sample	$125	$750
Features	De novo sequencing small genomes with BAC–BAC	De novo sequencing small genomes, resequencing and correcting sequence			De novo sequencing complex genomes	Order assembled contigs into scaffolds	De novo sequencing complex genomes, filling gaps and improving assembly	

BAC: bacterial artificial chromosomes.

**Table 2:** Examples of genome sequencing and assembly by long reads.

Species	Mean Subread Length	Number of Reads	Coverage of SMRT	Genome Size (Mb)	Contig N50 (Mb)	Assembly
*Utricularia gibba*	10,385	702,640	88	82	3.4	HGAP
*Oropetium thomaeum*	12,872	1,400,150	72	245	2.4	HGAP
*Chenopodium quinoa*	12,444	6,037,280	100	1500	1.7	SMRT-make
*Zea mays*	11,700	NA	65	2300	1.1	PBcR; Falcon
*Helianthus annuus*	10,300	32,000,000	102	3300	NA	PBcR

SMRT: Single Molecule Real-Time; HGAP: Hierarchical Genome Assembly Process; PBcR: PacBio Corrected Reads Hierarchical Assembly Pipeline; NA: not available.

**Table 3:** Comparison of the reference maize genome assembled by different sequencing platforms.

Assembly Parameters	Version 3	Version 4
Platform	Sanger and 454	PacBio and Bionano
Contig #	140,000	2958
Contig N50	19 Kb	1180 Kb
Scaffold #	61,161	625
Scaffold N50	76 Kb	9.5 Mb
Centromeres	Partial	Yes
Telomeres	Partial	Yes
Gap	10% missing	3% missing
